# Selenium Alleviates Porcine Nephrotoxicity of Ochratoxin A by Improving Selenoenzyme Expression *In Vitro*

**DOI:** 10.1371/journal.pone.0119808

**Published:** 2015-03-24

**Authors:** Fang Gan, Hongxia Xue, Yu Huang, Cuiling Pan, Kehe Huang

**Affiliations:** College of Veterinary Medicine, Nanjing Agricultural University, Nanjing, Jiangsu Province, China; Institut National de la Santé et de la Recherche Médicale (INSERM), FRANCE

## Abstract

Ochratoxin A (OTA), a mycotoxin, is a potent nephrotoxin in humans and animals. Selenium (Se) is an essential micronutrient for humans and animals, and plays a key role in antioxidant defense. To date, little is known about the effect of Se on OTA-induced nephrotoxicity. In this study, the protective effects of selenomethionine against OTA-induced nephrotoxicity were investigated using the porcine kidney 15 (PK15) cells as a model. The results showed that OTA induced nephrotoxicity in a dose-dependent manner. Se at 0.5, 1, 2 and 4 μM had significant protective effects against OTA-induced nephrotoxicity. Furthermore, selenomethionine enhanced the activity and mRNA and protein expression of glutathione peroxidase 1 (GPx1), mRNA expression of GPx4, and mRNA expression of thioredoxin reductase 1 in the presence and absence of OTA. Among them, promoting effect of selenomethionine on GPx1 was maximal. Knock-down of GPx1 by using a GPx1-specific siRNA eliminated the protective effects of selenomethionine against OTA-induced nephrotoxicity. The results suggest that selenomethionine alleviates OTA-induced nephrotoxicity by improving selenoenzyme expression in PK15 cells. Therefore, selenomethionine supplementation may be an attractive strategy for protecting humans and animals from the risk of kidney damage induced by OTA.

## Introduction

Ochratoxins are secondary metabolic products of several species of Aspergillus and Penicillium [1]. Among ochratoxins, ochratoxin A (OTA) shows the highest toxicity. Because of its widespread presence in food and feeds, such as corn silage, barley, oats, rye, and wheat [2], animals and humans are frequently exposed to OTA, which causes their accumulation in animal meat, human blood, and breast milk [3]. OTA has been identified as a nephrotoxin, hepatotoxin, immunotoxin, and genotoxin in animals and humans [4, 5]. The primary target organ of OTA is the kidney. It is suspected that OTA causes human Balkan endemic nephropathy, a disease that is reported to occur in some individuals of Yugoslavia, Bulgaria, and Romania [6, 7]. Samples from a group of Algerian patients who suffered from different nephropathies have shown greater OTA-positivity (95%) compared with the general population (67%) [8]. The adverse effects of OTA on humans and animals include loss of life, increased veterinary care costs, and reduced livestock production [9, 10].

The precise mechanisms underlying OTA toxicity have not been clearly determined. However, the ability of OTA to generate the reactive oxygen species (ROS) and disturb antioxidant enzymes may be the key to the lipid, protein, and DNA damages as well as the cell death [11–13]. Arbillaga et al. found that OTA causes an increase in the ROS production, leading to DNA damage and cytotoxicity [12]. Furthermore, these authors found that N-acetylcysteine (NAC) pretreatment protected the renal proximal tubular cells from damage induced by OTA. Yang et al. found that NAC reversed the effects of OTA on the increase in ROS production and the decrease in cell viability in human embryonic kidney cells [14]. Kumar et al. reported that OTA-induced apoptosis and oxidative stress in rabbit kidneys, leading to the pathogenesis of nephrotoxicity [15]. Klaric et al. showed that OTA induced cytotoxicity and apoptosis in porcine kidney 15 (PK15) cells [16]. Together, these findings clearly suggested that OTA-induced nephrotoxicity is related to oxidative damage.

Selenium (Se), an antioxidant trace element for humans and animals, plays a key role in redox regulation and antioxidant defense [17, 18]. The biological effects of Se are due to its incorporation into the selenocysteine and further into the selenoproteins [19]. Selenoproteins, such as Se-dependent glutathione peroxidase (GPx) and thioredoxin reductase (TR) are involved in the cellular antioxidant defense system[20]. Among these selenoproteins, GPx plays an important role in the elimination of hydrogen peroxide using GSH, whereas TR is critical for many cellular processes involving thiol-dependent redox mechanisms. It has been reported that Se supplementation increases GPx1 activity and GSH levels, enhances GPx1, GPx4, and TR1 gene expression, reduces ROS levels [21], and attenuates caspase-3 activity [22]. The effects of organic Se on the antioxidant defense are better than that of inorganic Se [23]. Furthermore, it has been shown that Se alleviates cytotoxicity induced by AFB1 and oxidative stress in Madin–Darby canine kidney cells and T-2-induced toxicity in chicken [24, 25]. However, the effect of Se on OTA-induced toxicity remains largely unknown.

Since OTA-induced nephrotoxicity is associated with oxidative damage [15, 16] and previous work has indicated that Se plays a key role in antioxidant defense [17, 18]. Therefore, we hypothesized that Se could protect from OTA-induced nephrotoxicity by improving selenoenzyme expression. The objective of this study was to investigate the protective effects of selenomethionine (organic Se) against OTA-induced nephrotoxicity and their antioxidant mechanisms in PK15 cells.

## Material and Methods

### Cell culture

The porcine kidney epithelial cells PK15 was obtained from the China Institute of Veterinary Drug Control. The cells were maintained in Dulbecco’s minimal Eagle’s medium (DMEM, Invitrogen, USA) supplemented with 5% heat-inactivated fetal bovine serum (FBS), 1% penicillin, and 1% streptomycin at 37°C in a humidified atmosphere of 5% CO_2_. Mycotoxin stock solution (2 mg/mL) used in the experiments was prepared by dissolving OTA in DMSO (100%). Final concentrations of mycotoxin were obtained by dilution in the culture medium. DMSO was added to cells without OTA treatment in the final concentration of 0.2%.

### Assessment of cytotoxicity by MTT

For the assessment of cytotoxicity, PK15 cells were cultured in 96-well plates at a density of 4 × 10^3^ cells/well with corresponding treatment. Following this, cells were assessed by the 3-(4,5-Dimethylthiazol-2-yl)-2,5-diphenyltetrazoliumbromide assay (Sigma, USA). Absorbance was measured at 490 nm and at a secondary wavelength of 650 nm. The results were expressed as percentage of control values.

### Assessment of lactate dehydrogenase (LDH) activity

For the assessment of LDH activity, PK15 cells were cultured in 12-well plates at a density of 8 × 10^4^ cells/well with corresponding treatment. After the treatment, the culture medium was collected in 1.5-mL Eppendorf tubes and centrifuged at 12,000 rpm for 15 min at 4°C. The supernatants were stored at −20°C until analysis. LDH activity was determined as described previously [26]. One unit of enzyme activity was defined as equivalent to 1 μmol of reduced nicotinamide adenine dinucleotide oxidized per minute. The data were expressed as percentage of the control values.

### Assessment of caspase-3 activity

For the assessment of caspase-3 activity, PK15 cells were cultured in 6-well plates at a density of 2 × 10^5^ cells/well with corresponding treatment. At the end of the treatment, caspase-3 activity in PK15 cells was assessed using the colorimetric assay kit (KeyGEN, China) according to the manufacturer’s instructions. Briefly, cells were washed twice with ice-cold PBS and lysed by incubating in 100 μL of lysis buffer on ice for 30 min. The lysate was centrifuged at 12,000 rpm for 5 min at 4°C and the supernatant was collected. Protein concentration in the supernatant was determined using the bicinchonininc acid (BCA) protein assay kit (Beyotime, China). Following this, 150 μg of each sample was incubated with caspase-3 substrate (20 mmol/dm3Ac-DEVD-pNA) for 4 h at 37°C in the dark. Then, the absorbance of the reaction mixture at 405 nm was measured using a microplate reader (Bio-RAD). Caspase-3 activity was calculated as OD (inducer)/OD (negative control) and expressed as percentage of control values.

### Assessment of annexin V binding by flow cytometry

For the assessment apoptosis by annexin V staining, PK15 cells were cultured at a density of 2 × 10^5^ cells/well in 6-well plates with corresponding treatment and apoptosis was monitored by annexin V/PI (BD Pharmingen) method as described previously [27] with minor modification. Briefly, after removing the culture medium, cells were washed two times with PBS, then resuspended in 100 μL of 1× binding buffer, and incubated with 5 μL of annexin V and 5 μL of PI at 25°C in the dark for 15 min. For flow cytometric analysis, the reaction volume was raised to 500 μL by adding binding buffer.

### Determination of intracellular ROS by flow cytometry

For the determination of ROS, PK15 cells were cultured at a density of 2 × 10^5^ cells/well in 6-well plates with corresponding treatment and the intracellular ROS was measured as described previously [28] using the oxidation-sensitive dye 2′,7′-dichlorofluorescein diacetate (DCFH-DA). Briefly, after removing the culture medium, cells were washed three times with serum-free DMEM and incubated with 10 μM of DCFH-DA for 30 min at 37°C. Subsequently, the cells were washed three times with serum-free DMEM and re-suspended in PBS. Intracellular ROS level was expressed as % of the control values.

### Determination of reduced glutathione

For assessing the levels of reduced glutathione, PK15 cells were cultured at a density of 2 × 10^5^ cells/well in 6-well plates with corresponding treatment. Following this, the cells were collected, sonicated (SonicsVCX105, USA) in ice-cold PBS, centrifuged at 12,000 rpm for 20 min to remove debris, and the supernatant was collected. The reduced GSH was determined spectrophotometrically by measuring the absorbance at 412 nm after reaction with 5, 5′-dithiobis (2-nitrobenzoicacid) as described previously [21] using commercially available kits (Jiancheng, China). Total protein concentration was determined using the BCA protein assay kit (Beyotime, China). The data were expressed as nanomoles of GSH per milligram of protein.

### Glutathione peroxidase 1 (GPx1) activity assay

For GPx1 activity assay, PK15 cells were cultured at a density of 6 × 10^4^ cells/well in 12-well plates with corresponding treatment. The activity of GPx1 was measured with tert-butyl hydroperoxide (t-Bu-OOH) as substrate as described previously [29]. Briefly, cell extracts prepared by sonication (SonicsVCX105, USA) in ice-cold PBS was centrifuged at 12,000 rpm for 20 min to obtain the supernatant. Then, 50 μL of the supernatant was added to 1900 μL of the reaction mixture [50 mM Tris-HCl, pH 7.5, 2 mM EDTA, 2 mM GSH, 1 mM NaN3, 0.1 mM NADPH, and 0.9 U of GSH reductase (Sigma, USA)] and pre-incubated for 3 min at 25°C. Following this, 50 μL of t-Bu-OOH (8 mM) was added to the reaction mixture and the rate of NADPH oxidation was assessed spectrophotometrically by measuring the absorbance at 340 nm at 25°C for 5 min. The nonenzymatic reaction rate was determined by substituting water (serving as the blank) for the cell extracts. One unit of enzyme activity was defined as equivalent to 1 μmol of NADPH oxidized per minute under these conditions. GPx1 activity was expressed as % of the control values.

### Determination of selenoprotein mRNA levels by real-time PCR

Primers (**S1 Table**) for the analysis of three selenoprotein genes (GPx1, GPx4, and TR1) and 1 reference gene (β-actin) were designed using the Primer Premier Software (PREMIER Biosoft International, Palo Alto, CA, USA) based on known porcine sequences.

For gene expression analysis, PK15 cells were cultured at a density of 6 × 10^4^ cells/well in 12-well plates with corresponding treatment. Total RNA was extracted using the RNAiso Plus kit (TaKaRa, China) according to the manufacturer's protocols. Potential DNA contamination of the extract was eliminated using the DNA-Free kit (TaKaRa) and the RNA quality was assessed indirectly from the ratio OD_260_/OD_280_. First-strand cDNA was synthesized from 1 μg of total RNA using Oligo dT primers and M-MLV reverse transcriptase (TaKaRa, China) according to the manufacturer' instructions. Real-time PCR analysis of gene expression was carried out using the ABI Prism Step One Plus detection system (Applied Biosystems, USA). Typically, a total 25 μL of the reaction mixture contained 12.5 μL of 2× SYBR Green I PCR Master Mix (TaKaRa, China), 10 μL of cDNA, 1 μL of each primer (10 μM), and 0.5 μL of PCR-grade water. The following thermal cycling condition was used: a single step at 95°C for 30 s, followed by 40 cycles of 95°C for 5 s and 60°C for 30 s. A dissociation curve was run for each plate to confirm that a single PCR product was formed. A no-template control served as the negative control. The relative mRNA levels of selenoproteins were determined using the Δ cycle threshold (ΔCt) method with β-actin serving as the reference gene.

### Determination of GPx1 protein expression by western blot

For the assessment of protein expression, PK15 cells were cultured at a density of 2 × 10^5^ cells/well in 6-well plates with corresponding treatment. After the treatment, the cells were washed twice with 2 mL of ice-cold PBS and lysed with 80 μL of lysis buffer containing protease inhibitor (Beyotime, China). The lysate was sonicated (SonicsVCX105, USA) and the homogenate was centrifuged at 12,000 rpm for 20 min at 4°C. Following this, the supernatant was collected and used immediately or snap-frozen in liquid nitrogen. Protein concentration was determined using the BCA kit (Beyotime, China). Forty μg of protein was diluted in sample loading buffer and heated at 95°C for 5 min. The denatured proteins were separated by 12% sodium dodecyl sulfate-polyacrylamide gel electrophoresis (SDS–PAGE), and transferred onto polyvinylidene difluoride (PVDF) membranes (Bio-Rad Trans-Blot SD). The membranes were first incubated for 40 min at room temperature in Tris-buffered saline (TBS) containing 5% BSA and 0.1% Tween 20 (TBST) and then incubated overnight in TBST containing specific primary antibodies [goat anti-GPx1 (Santa Cruz Biotechnology), diluted 1/500; rabbit anti-Actin (Cell Signaling pathway), diluted 1/1000]. After three washes in TBST, the membranes were incubated with HRP-conjugated secondary antibody [HRP-conjugated anti-goat (Santa Cruz Biotechnology) for GPx1, or polyclonal HRP-conjugated anti-rabbit (Sigma) for Actin] for 1h at room temperature. The membranes were then washed three times for 5 min and the immunocomplexes were visualized with the help of the standard enhanced chemiluminescence system (Bio-Rad).

### Small interfering RNA (siRNA) transfection

Using the sequence of Sus scrofa GPx1 mRNA (GenBank Accession No. NM_214201.1), a GPx1-specific siRNA was designed with the help of Invitrogen BlockiT RNAi designer. The sequence of the GPx1-specific siRNA was 5′- GGGACUACACCCAGAUGAAtt-3′. The sequence of the control siRNA was 5′-UUCUCCGAACGUGUCACGUtt-3′. The two double-stranded RNAs were synthesized by Invitrogen. Before use, the duplexes were re-suspended in RNA-free water at a concentration of 20 μM. PK15 cells were seeded in 96-, 12-, and 6-well plates at a density of 4 × 10^3^, 8 × 10^4^, and 2 × 10^5^ cells/well, respectively, and cultured overnight at 37°C in the absence of antibiotics in DMEM containing 8% FBS. When the cultures reached 30–50% confluence, the cells were transfected with siRNA using the X-tremeGene siRNA transfection reagent (Roche, USA) according to the manufacturer’s protocols. Typically, the transfection reagent and siRNA (5:1) were added to each well and incubated for 5 h. The cells were then washed with DMEM and cultured in DMEM containing 4% FBS for various treatments.

### Statistical analysis

Data were statistically analyzed by a one-way analysis of variance (ANOVA), followed by Duncan’s multiple range tests to analyze the means. All statistical analyses were performed with the help of the SPSS for Windows (version 17.0) program. Results are presented as the mean ± standard error (SE). *P* < 0.05 was considered statistically significant.

## Results

### Cytotoxic effects of OTA or Se on PK15 cells

**Fig 1:**
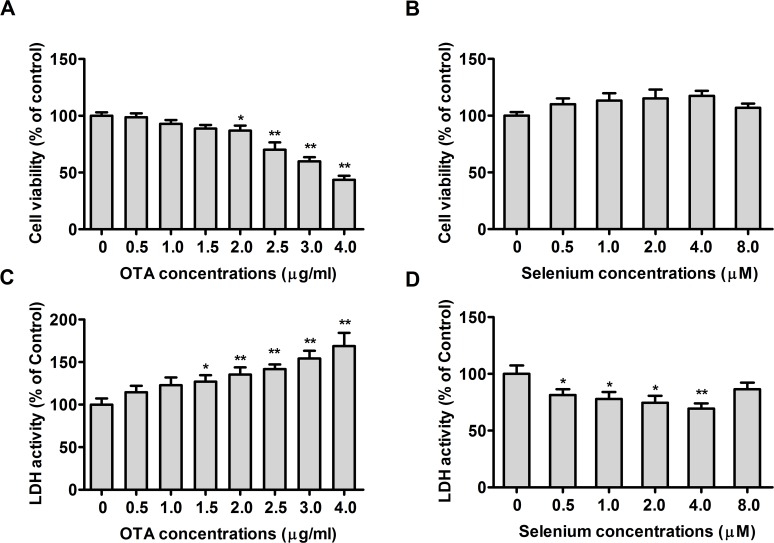
Effects of ochratoxin A (OTA) or selenium on cell viability and LDH activity in PK15 cells. Cell viability (A, B) and LDH activity (C, D) were assayed as described in the Materials and Methods section. Data are presented as mean ± SE (n = 4). Significance compared with control (0 μg/mL OTA or 0 μM Se), *P < 0.05 and **P < 0.01.

To assess the cytotoxic effects of selenomethionine (organic Se) or OTA on PK15 cells, cells were incubated with 0, 0.5, 1.0, 1.5, 2.0, 2.5, 3.0, or 4.0 μg/mL of OTA for 48 h or with 0, 0.5, 1, 2, 4, or 8 μM of Se (from selenomethionine) for 72 h, following which cell viability and LDH activity were measured. As shown in **Fig. 1**, 2.0 to 4 μg/mL of OTA significantly reduced the viability of PK15 cells (*P* < 0.05) in a dose dependent manner. This effect of OTA on cell viability was extremely significant (*P* < 0.01) at 2.5 μg/mL. 1.5 to 4 μg/mL of OTA significantly increased LDH activity in PK15 cells **(Fig. 1C)** in a dose dependent manner (*P* < 0.05). This effect of OTA on LDH activity was extremely significant at 2.0 μg/mL concentration (*P* < 0.01). However, the effect of Se on the viability of PK15 cells was limited and insignificant (**Fig. 1B)**. Except at 8 μM of Se, Se treatment significantly reduced LDH activity in PK15 cells (**Fig. 1D)** (*P* < 0.05). Therefore, 2.5 μg/mL of OTA and 0.5, 1, 2 and 4 μM of Se from selenomethionine were used in subsequent experiments.

### Protective effects of selenomethionine against OTA-induced cytotoxicity

**Fig 2:**
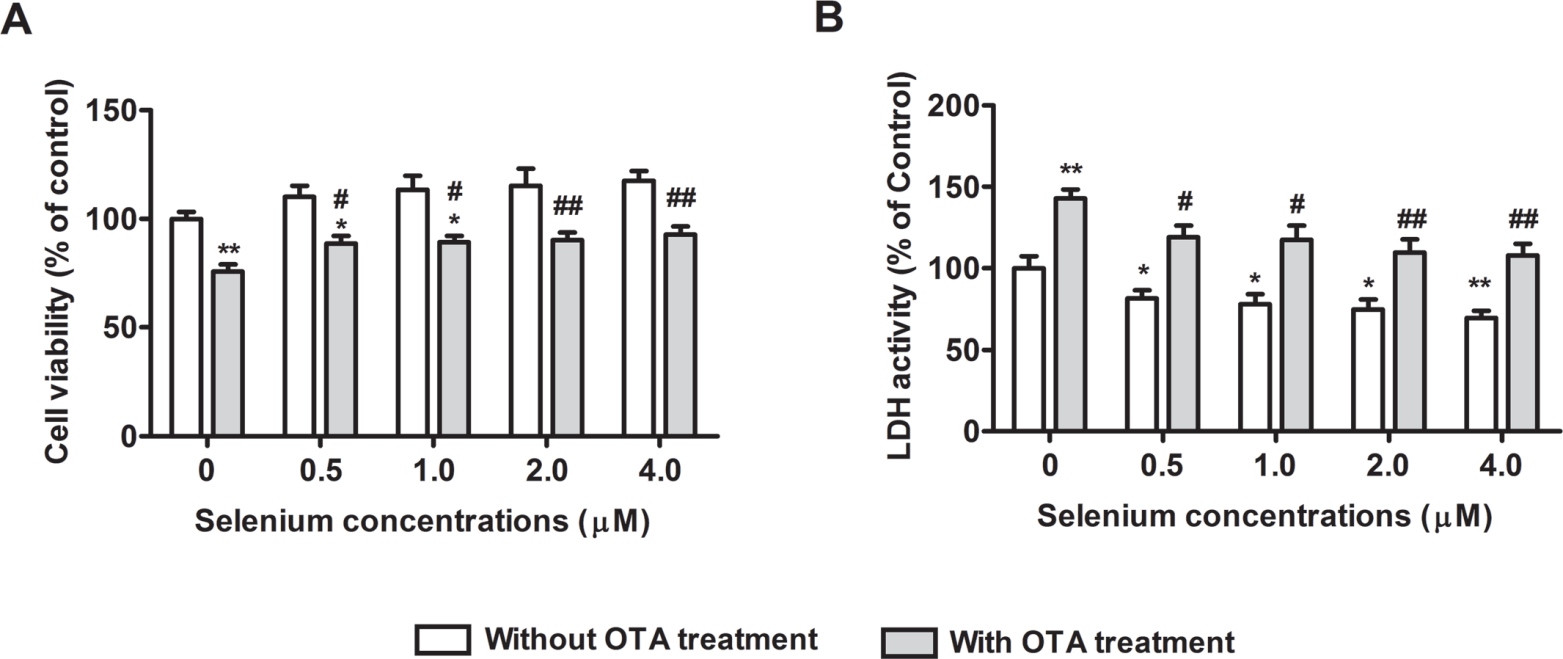
Protective effects of selenomethionine against OTA-induced cytotoxicity in PK15 cells. Cell viability (A) and LDH activity (B) were assayed as described in the Materials and Methods section. Data are presented as mean ± SE (n = 3). Significance compared with control (without OTA or selenomethionine), *P < 0.05 and **P < 0.01. Within the OTA treatment group, significance compared with the control cells without selenomethionine treatment, #*P* < 0.05 and ##*P* < 0.01.

To evaluate the protective effects of selenomethionine against OTA’s cytotoxicity, PK15 cells were cultured with 0, 0.5, 1, 2, or 4 μM of Se (from selenomethionine) for 24 h and then incubated in the presence or absence of 2.5 μg/mL of OTA and Se for a further 48 h. As shown in **Fig. 2**, OTA significantly reduced the cell viability (**Fig. 2A**) and increased LDH activity (**Fig. 2B**). Compared with OTA treatment controls, selenomethionine significantly reversed these OTA-induced changes in a dose dependent manner (*P* < 0.05). These results suggested that selenomethionine afforded significant protection against OTA-induced cytotoxicity.

### Protective effects of selenomethionine against OTA-induced apoptosis

**Fig 3:**
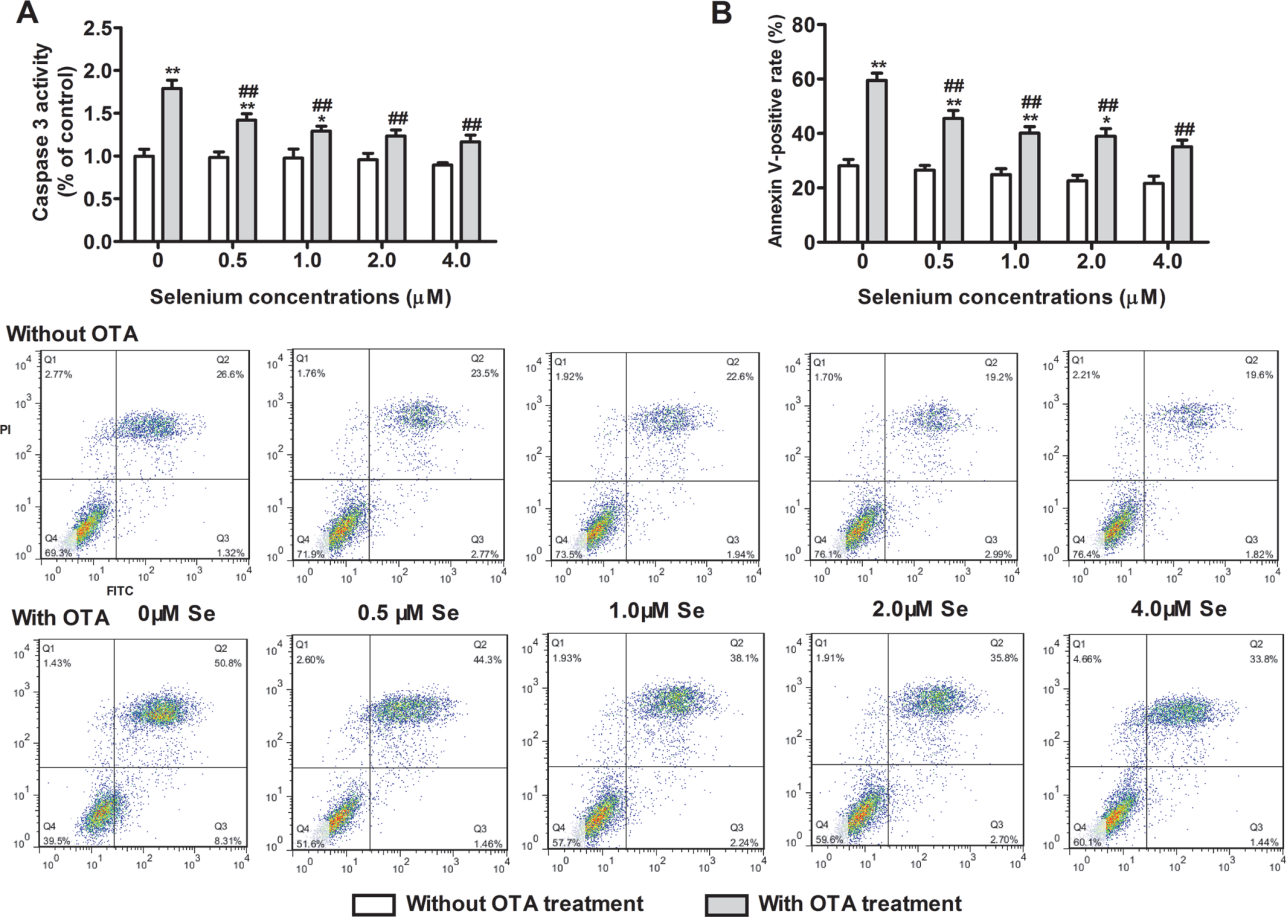
Protective effects of selenomethionine against OTA-induced apoptosis in PK15 cells. Caspase-3 activity (A) and annexin V-binding (B) were assayed as described in the Materials and Methods section. Data are presented as mean ± SE (n = 3). Significance compared with control (without OTA or selenomethionine), *P < 0.05 and **P < 0.01. Within the OTA treatment group, significance compared with the control cells without selenomethionine treatment, #*P* < 0.05 and ##*P* < 0.01.

To evaluate the protective effects of selenomethionine against OTA-induced apoptosis, PK15 cells were cultured with 0, 0.5, 1, 2, or 4 μM of Se (from selenomethionine) for 24 h and then incubated in the presence or absence of 2.5 μg/mL of OTA and Se for an additional 48 h. As shown in **Fig. 3**, OTA increased caspase-3 activity (**Fig. 3A**) and annexin V-binding in PK15 cells (**Fig. 3B**) (*P* < 0.05). Compared with OTA treatment controls, selenomethionine significantly reversed these OTA-induced changes in a dose dependent manner (*P* < 0.05). These results suggested that selenomethionine offered significant protection from OTA-induced apoptosis.

### OTA-induced cytotoxicity and apoptosis is associated with oxidative stress

**Fig 4:**
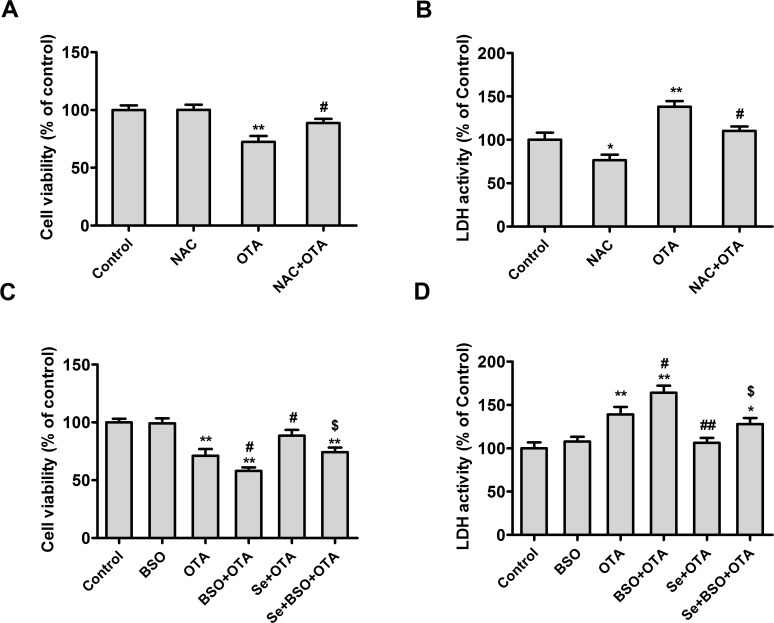
Effects of NAC or selenomethionine and/or BSO on OTA-induced cytotoxicity in PK15 cells. Cell viability (A, C) and LDH activity (B, D) were assayed as described in the Materials and Methods section. Data are presented as mean ± SE (n = 3). Significance compared with control, *P < 0.05 and **P < 0.01. Significance compared with OTA treatment, #*P* < 0.05 and ##*P* < 0.01. Significance compared with OTA and selenomethionine treatment, **$** *P* < 0.05 and **$$** *P* < 0.01.

**Fig 5:**
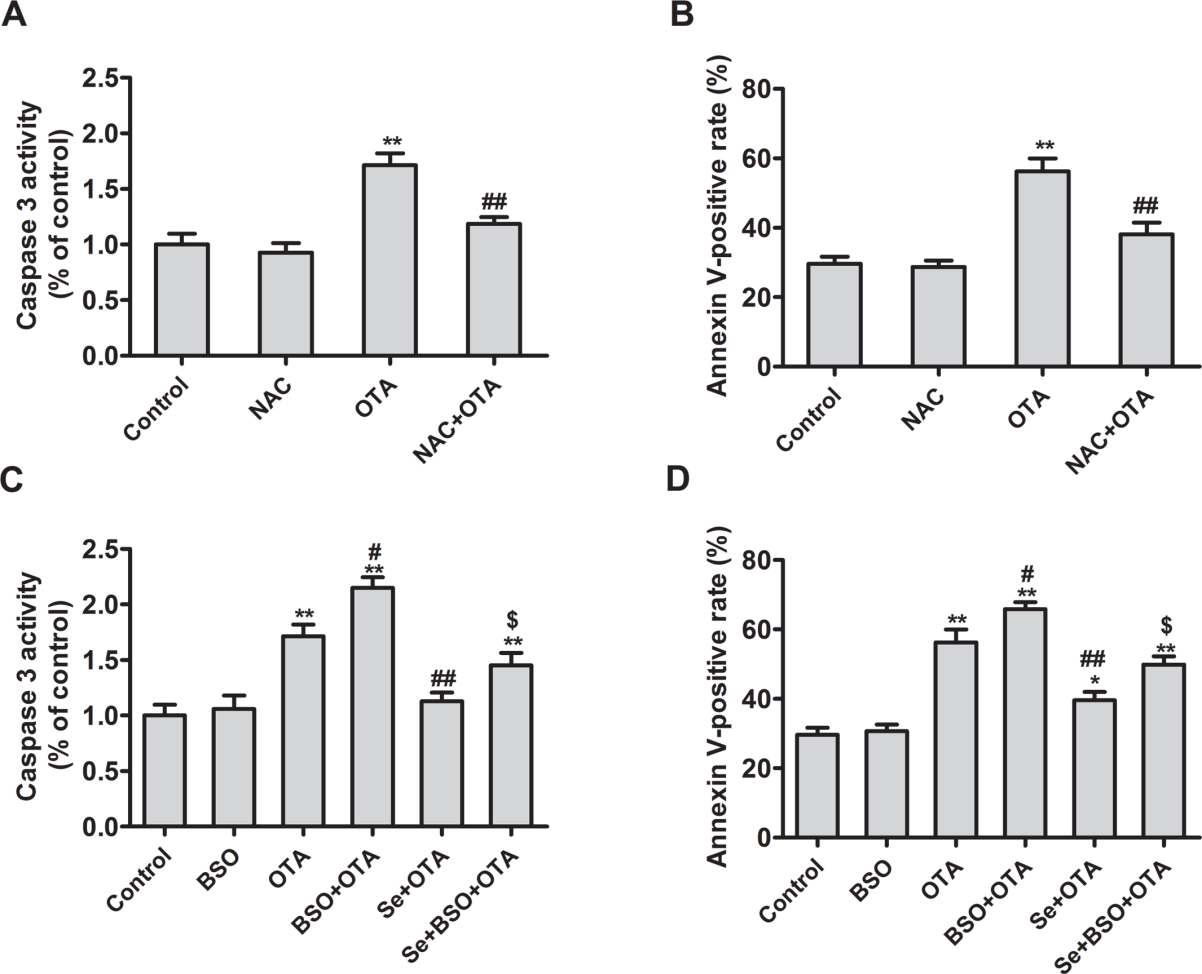
Effects of NAC or selenomethionine and/or BSO on OTA-induced apoptosis in PK15 cells. Caspase-3 activity (A, C) and annexin V-binding (B, D) were assayed as described in the Materials and Methods section. Data are presented as mean ± SE (n = 3). Significance compared with control, *P < 0.05 and **P < 0.01. Significance compared with OTA treatment, #*P* < 0.05 and ##*P* < 0.01. Significance compared with OTA and selenomethionine treatment, **$** *P* < 0.05 and **$$** *P* < 0.01.

To determine whether the OTA-induced nephrotoxicity was related to oxidative stress, PK15 cells were cultured with 50 μM of buthionine sulfoximine (BSO, a specific inhibitor of glutamate-cysteine ligase), 4 mM of NAC (a free radical scavenger), or 4 μM of Se (from selenomethionine) for 24 h and then incubated in the presence or absence of OTA and BSO/NAC/Se for an additional 48 h. As shown in **Figs. 4 and 5**, NAC alleviated the effect of OTA on cell viability (**Fig. 4A**) and LDH activity (**Fig. 4B**), caspase-3 activity (**Fig. 5A**), and annexin V-binding (**Fig. 5B**) (*P* < 0.05), compared with OTA group. In contrast, BSO promoted the effects of OTA on cell viability (**Fig. 4C**), LDH activity (**Fig. 4D**), caspase-3 activity (**Fig. 5C**), and annexin V-binding (**Fig. 5D**) (*P* < 0.05) compared with OTA group, and abrogated the protective effects of selenomethionine against OTA-induced cytotoxicity and apoptosis (*P* < 0.05). These results indicated that OTA-induced cytotoxicity and apoptosis was related to oxidative stress.

### Effects of selenomethionine supplementation on antioxidant capacity

**Fig 6:**
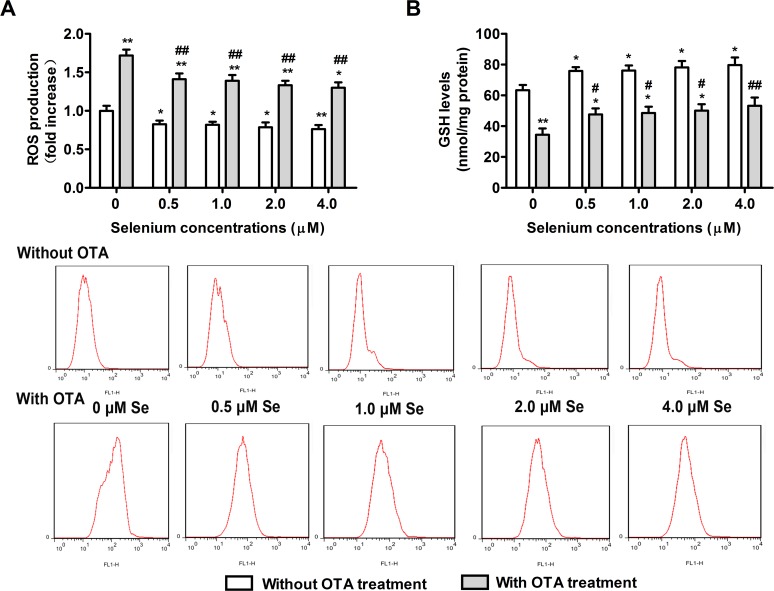
Effects of selenomethionine supplementation on antioxidant capacity of PK15 cells. Changes in ROS (A) and reduced GSH (B) levels were assayed as described in the Materials and Methods section. Data are presented as mean ± SE (n = 3). Significance compared with control (without OTA or selenomethionine), *P < 0.05 and **P < 0.01. Within the OTA treatment groups, significance compared with the control cells without selenomethionine treatment, #*P* < 0.05 and ##*P* < 0.01.

To examine whether the protective effects of selenomethionine against OTA-induced nephrotoxicity were related to its effects on antioxidant capacity, ROS and GSH levels in PK15 cells were measured. PK15 cells were cultured with 0, 0.5, 1, 2, or 4 μM of Se (from selenomethionine) for 24 h and then incubated in the presence or absence of 2.5 μg/mL of OTA for an additional 48 h. As shown in **Fig. 6**, OTA treatment increased ROS levels and reduced GSH levels in PK15 cells (*P* < 0.05). In contrast, Se supplementation reduced the ROS levels (**Fig. 6A**) and increased GSH levels (**Fig. 6B**) in PK15 cells in a dose dependent manner (*P* < 0.05). These results suggested that selenomethionine treatment augmented the cellular antioxidant capacity and abrogated OTA-induced oxidative stress, and that the protective effects of Se against OTA-induced nephrotoxicity were related to its effects on the cellular antioxidant capacity.

### Effects of selenomethionine supplementation on selenoenzyme expression

**Fig 7:**
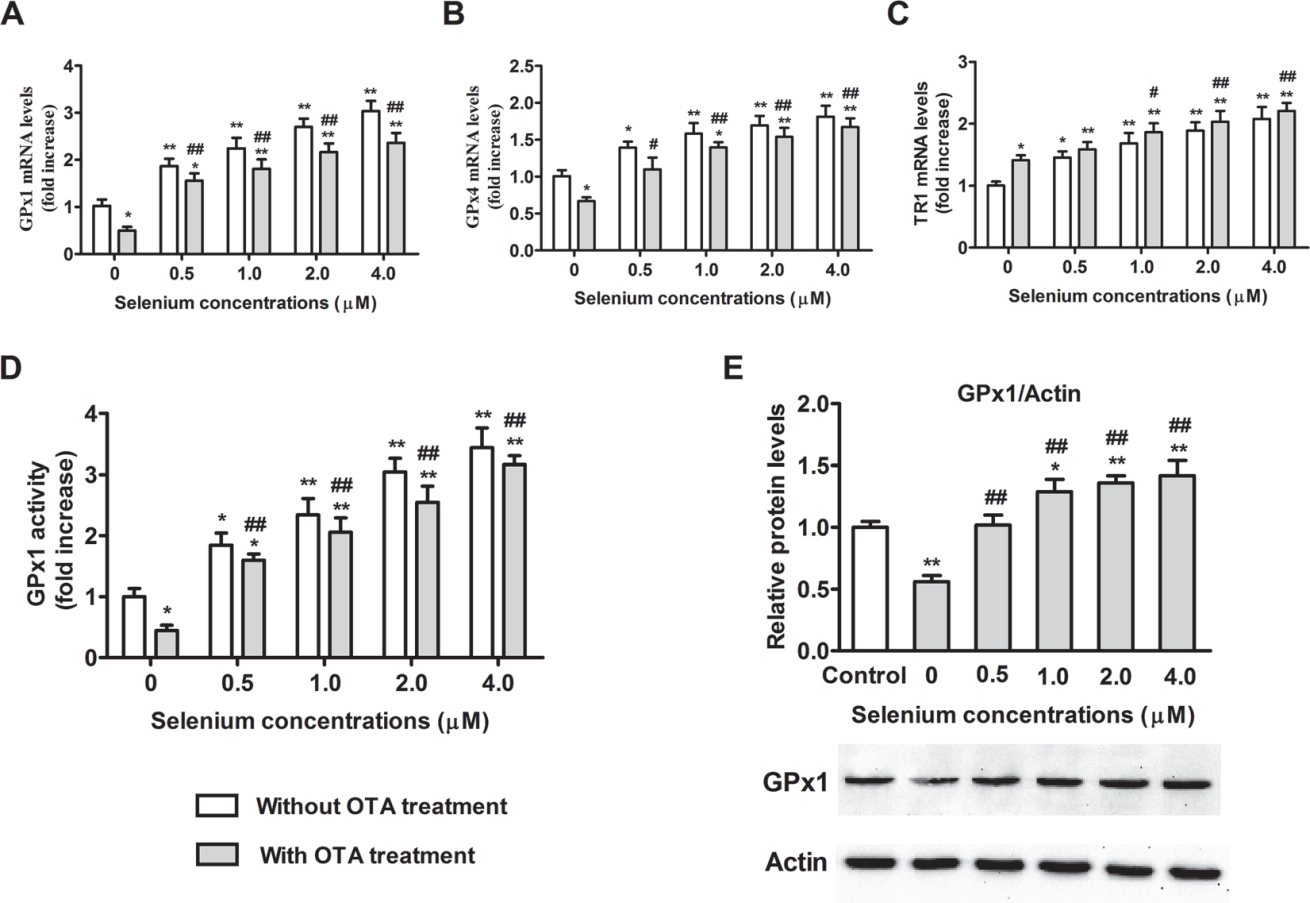
Effects of selenomethionine supplementation on selenoenzyme expression in PK15 cells. GPx1 (A), GPx4 (B), and TR1 (C) mRNA levels, GPx1 activity (D), and GPx1 protein expression (E) were assayed as described in the Materials and Methods section. Data are presented as mean ± SE (n = 3). Significance compared with control (without OTA or selenomethionine), *P < 0.05 and **P < 0.01. Within the OTA treatment group, significance compared with the control cells without selenomethionine treatment, #*P* < 0.05 and ##*P* < 0.01.

We investigated the effects of selenomethionine on selenoenzyme expression. PK15 cells were cultured with 0, 0.5, 1, 2, or 4 μM of Se (from selenomethionine) for 24 h and then incubated in the presence or absence of 2.5 μg/mL of OTA for an additional 48 h. As shown in **Fig. 7**, OTA treatment led to 52.2% reduction in GPx1 mRNA levels (**Fig. 7A**), 55.7% reduction in GPx1 activity (**Fig. 7D**), 43.8% reduction in GPx1 protein expression (**Fig. 7E**), 33.0% reduction in GPx4 mRNA levels (**Fig. 7B**), and a 41.2% increase in TR1 mRNA levels (**Fig. 7C**) (*P* < 0.05) compared with control group. Selenomethionine significantly increased these selenoenzyme expression both in OTA-treated and vehicle-treated cells. GPx1 mRNA expression showed the greatest change in response to 0.5, 1, 2, and 4 μM of Se from selenomethionine treatment, with fold increases of 1.87, 2.24, 2.70, and 3.04, respectively (*P* < 0.05) (**Fig. 7A**).

### Effect of GPx1 knockdown on GPx1 expression in PK15 cells

**Fig 8:**
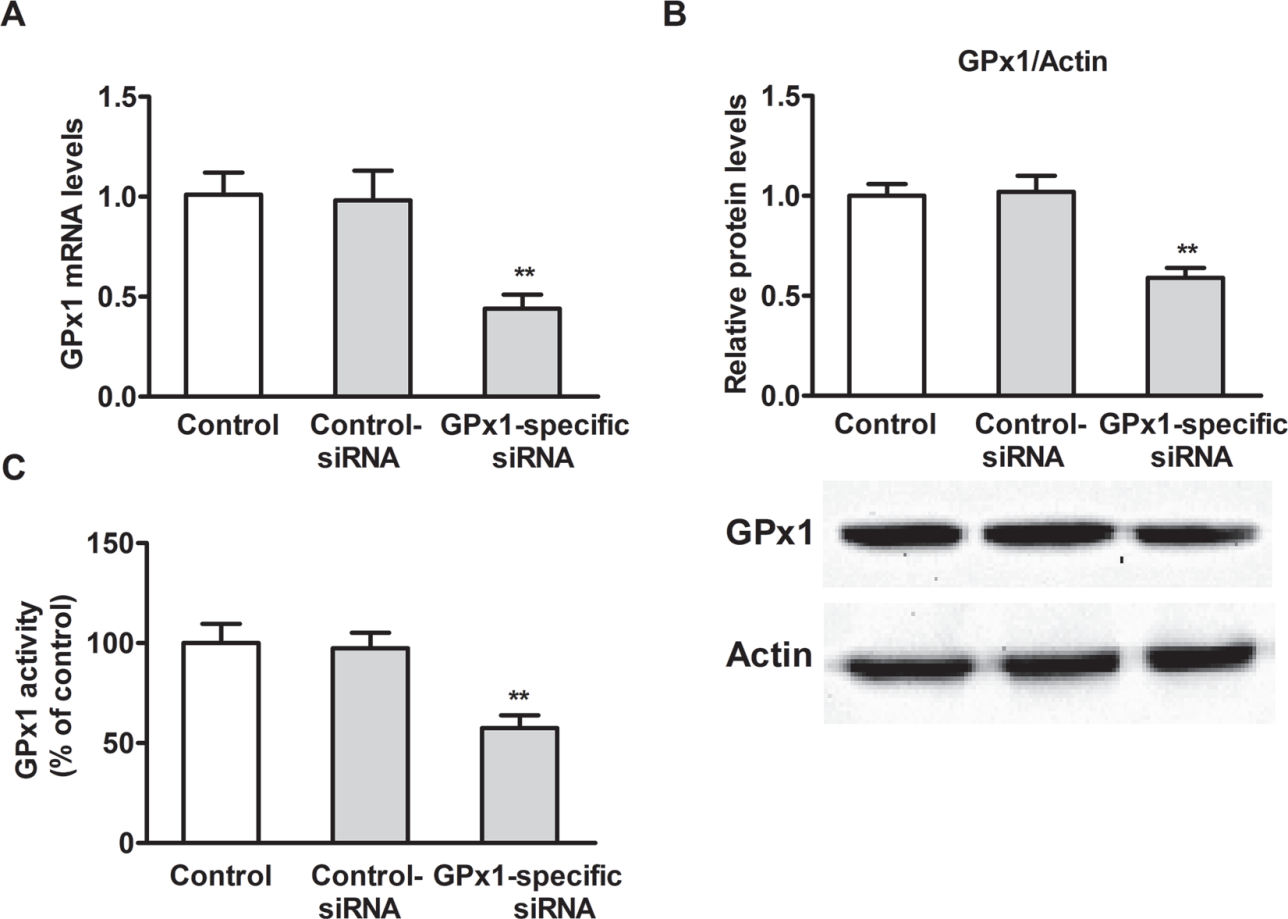
Effect of GPx1 knock-down on GPx1 expression in PK15 cells. GPx1 mRNA levels (A), GPx1 protein levels (B), and GPx1 activity (C) were assayed. Data are presented as mean ± SE (n = 3). Significance compared with control, *P < 0.05 and **P < 0.01.

To evaluate the extent of GPx1 knockdown, PK15 cells were cultured overnight with 4 μM of Se (from selenomethionine) and then transfected with GPx1-specific siRNA or control siRNA. As shown in **Fig. 8**, transfection of PK15 cells with GPx1-specific siRNA resulted in significant reduction in GPx1 mRNA (**Fig. 8A**) (*P* < 0.05) and protein levels (**Fig. 8B**) (*P* < 0.05) as well as GPx1 activity (**Fig. 8C**) (*P* < 0.05).

### Effects of selenomethionine against OTA-induced cytotoxicity and apoptosis in GPx1-knockdown PK15 cells

**Fig 9:**
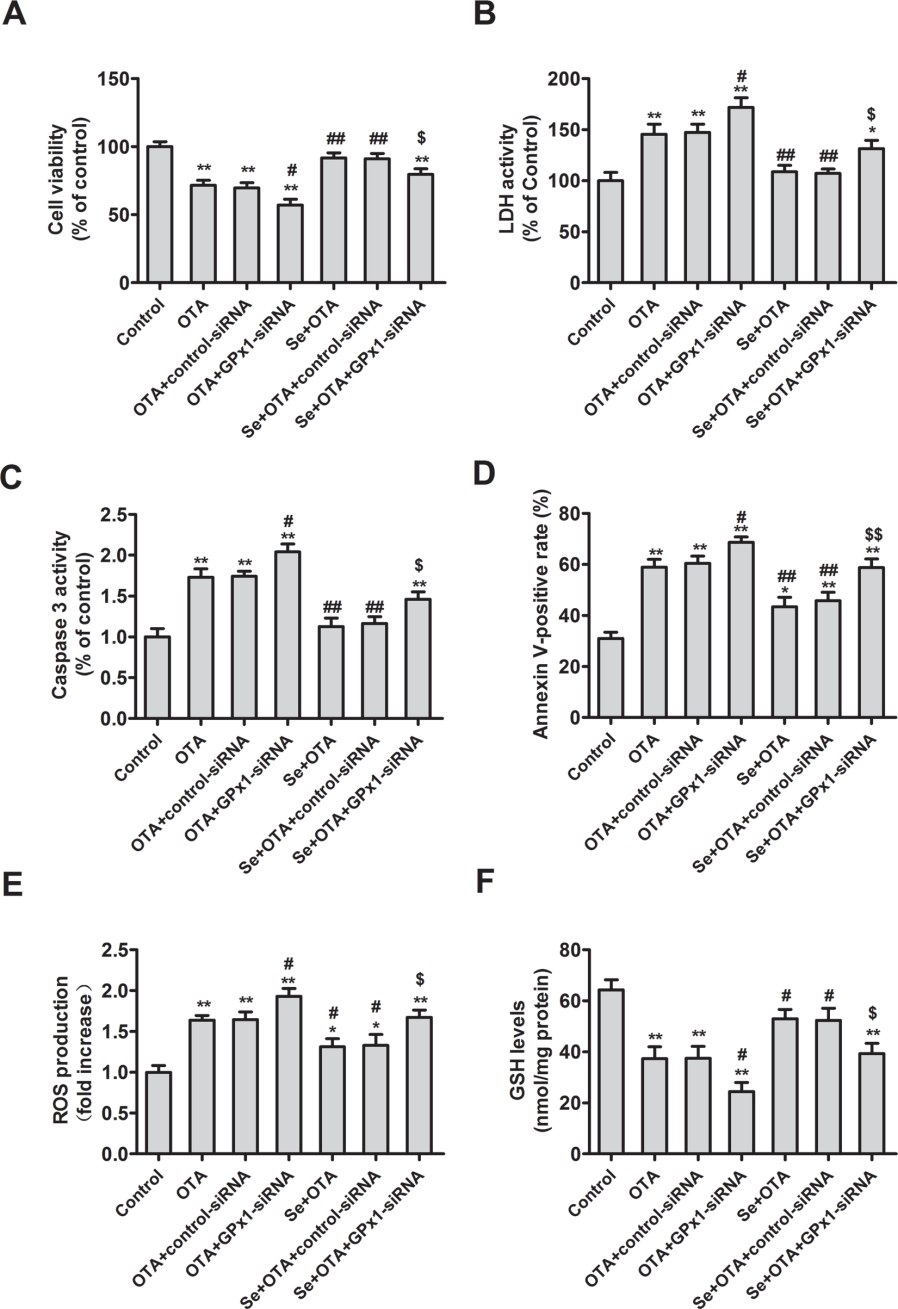
Effects of selenomethionine on OTA-induced cytotoxicity and apoptosis in GPx1-knockdown PK15 cells. Cell viability (A), LDH activity (B), caspase-3 activity (C), annexin V-binding (D), ROS production (E), and GSH levels (F) were assayed. Data are presented as mean ± SE (n = 3). Significance compared with control, *P < 0.05 and **P < 0.01. Significance compared with OTA treatment, #*P* < 0.05 and ##*P* < 0.01. Significance compared with selenomethionine and OTA treatment, **$** *P* < 0.05 and $$*P* < 0.01.

To assess whether GPx1 played a key role in the protective effects of selenomethionine against OTA-induced cytotoxicity and apoptosis, PK15 cells were cultured overnight and then transfected with GPx1-specific or control siRNA. After 5 h of transfection, the medium was removed and the transfected cells were cultured with or without 4 μM of Se for 24 h. Following this, the cells were incubated with 2.5 μg/mL of OTA for an additional 48 h. As shown in **Fig. 9**, GPx1-specific siRNA promoted the effects of OTA on cell viability **(Fig. 9A)**, LDH activity **(Fig. 9B)**, caspase-3 activity **(Fig. 9C)**, annexin V-binding **(Fig. 9D)**, ROS production **(Fig. 9E)**, and GSH levels **(Fig. 9F)**. Additionally, GPx1-specific siRNA abrogated the effects of Se on OTA-induced changes in cell viability, LDH activity, caspase-3 activity, annexin V-binding, GSH and ROS levels (*P* < 0.05). Together, these results suggested that GPx1 is necessary for the protective effects of selenomethionine against cytotoxicity and apoptosis induced by OTA in PK15 cells.

## Discussion

It has been reported that mycotoxins such as AFB1, T-2, and OTA induce toxicity and oxidative stress [16, 24, 30, 31]. It is also known that Se could relieve the oxidative stress induced by AFB1 in MDCK cells [24] or T-2 in chicken [25] and apoptosis in rats [22], but its effects on OTA-induced nephotoxicity haven’t been reported until now. Therefore, we investigated the protective effects of Se against OTA-induced cytotoxicity and apoptosis in PK15 cells. Our results showed that OTA-induced cytotoxicity and apoptosis were related to oxidative damage and that selenomethionine supplementation protected PK15 cells against the OTA-induced cytotoxicity and apoptosis. Furthermore, we showed that selenomethionine supplementation alleviated the OTA-induced cytotoxicity and afforded protection from apoptosis by improving selenoenzyme expression, particularly the expression of GPx1. Since OTA is frequently found in feed and food, selenium supplementation may be an attractive strategy to protect humans and animals from the adverse effects of OTA.

The present results showed that the cytotoxic effects of OTA on PK15 cells were dose dependent. This was consistent with previous reports [16]. In the present study, we used selenomethionine, an organic Se source with lower toxicity than sodium selenite [21, 32]. At the concentrations used, Se had no toxic effect on PK15 cells. Our results showed that selenomethionine was effective in protecting cells from OTA-induced loss of cell viability and increase in LDH activity. These results suggested that Se supplementation significantly protected PK15 cells from OTA-induced cytotoxicity. Our results are consistent with that of previous studies, which found that Se significantly reduced T-2-induced cytotoxicity in human hepatoma cell line [33] and protected cells from AFB1-induced cytotoxicity in Chinese hamster ovary cells [34].

Activation of caspase-3 is an early hallmark during apoptosis [35]. Previous studies have shown that OTA increased caspase-3 activity in human tubular kidney cells [36] and annexin-V-binding in human vascular endothelial cells [37] and in rats [38]. In addition, it has been reported that Se could protect rats from traumatic brain injury-induced apoptosis [22]. However, up to now, there has no reports of the effects of Se on apoptosis induced by OTA in PK15 cells. Consistent with the previous reports [36–38], our results showed that OTA treatment significantly increased caspase-3 activity and annexin-V-binding in PK15 cells. Our results also showed that pre-treatment of PK15 cells with selenomethionine significantly prevented the OTA-induced changes in caspase-3 activity and annexin-V-binding. These results clearly suggested that Se offered significant protection from OTA-induced apoptosis.

It has been reported that ROS is generated endogenously during cellular metabolism or exogenously during oxidative stress [39], and OTA treatment induced GSH depletion and ROS production [2, 11, 40]. To evaluate whether OTA-induced cytotoxicity and apoptosis are associated with oxidative stress in PK15 cells, NAC (a free radical scavenger) were used in the present study. Our results showed that NAC prevented the nephrototoxic effects of OTA in PK15 cells, consistent with the results of previous studies that NAC significantly reversed cytotoxicity induced by OTA in HEK-293 cells [14] and induced by BSO in the DRG neurons [41]. In addition, the finding in the present study that OTA treatment for 48 h led to a significant increase in ROS levels and reduction in GSH levels are in agreement with the results of previous studies that OTA exposure led to increase of intracellular ROS production in human hepatoma cells [42] and decrease of GSH levels in PK15 cells [43]. The reduction in GSH levels may have been caused by its over-utilization for the suppression of ROS [44]. Our results strongly suggested that OTA-induced cytotoxicity and apoptosis was related to intracellular oxidative stress in PK15 cells.

Since the nephrotoxicity of OTA are associated with intracellular oxidative stress and the previous studies have shown that selenomethionine alleviates oxidative stress [21, 24]. Therefore, we hypothesized that selenomethionine might protect PK15 cells against OTA-induced cytotoxicity and apoptosis by improving the intracellular redox statues. Consistent with the results of previous studies of NIH/3T3 cells [45] and rat neurons [41, 46], our results showed that BSO augmented OTA-induced cytotoxicity and apoptosis and eliminated the protective effects of selenomethionine against OTA-induced cytotoxicity and apoptosis in PK15 cells. The present results also showed that pre-treatment with selenomethionine significantly increased GSH levels and reduced ROS levels in PK15 cells in the presence or absence of OTA. Furthermore, our results showed that selenomethionine blocked OTA-induced ROS production and GSH depletion in PK15 cells in a dose-dependent manner. These results are consistent with that of a previous study, which found that selenomethionine treatment lowered the total amount of ROS generated by As (III) treatment in HEK293 human kidney cells [47]. Together, the present results strongly suggested that selenomethionine protected PK15 cells against OTA-induced cytotoxicity and apoptosis by improving cellular antioxidant capacity.

In the present study, we observed that OTA significantly reduced GPx1 activity, GPx1 mRNA and protein expression, and GPx4 mRNA expression, and increased TR1 mRNA expression. The reduced expression of GPx1 and GPx4 mRNAs may have been due to the increased oxidative damage, consistent with the previous studies in bovine peripheral blood mononuclear cells and MDCK cells of AFB1[24, 48]. The increase in TR1 mRNA expression likely reflected the need for maintaining ROS homeostasis [49]. We found that selenomethionine pretreatment significantly increased GPx1 activity, GPx1 mRNA and protein expression, GPx4 mRNA expression, and TR1 mRNA expression, and GPx1 expression has maximum increase among these selenoenzymes, consistent with our previous study [21]. That the effect of Se on GPx1 mRNA expression was the highest suggested that GPx1 is highly sensitive to Se intake [50]. Therefore, we used GPx1-specific siRNA to knockdown GPx1 in PK15 cells. The present results showed that GPx1-specific siRNA partially blocked the protective effects of selenomethionine against OTA-induced cytotoxicity and apoptosis. This is the first report describing the key role of GPx1 in Se protecting cells from OTA-induced cytotoxicity and apoptosis. Together, the present results suggest that selenomethionine exerts its protective effects against OTA-induced cytotoxicity and apoptosis by improving selenoenzyme expression, especially the expression of GPx1.

## Conclusion

In conclusion, the present results demonstrate the involvement of oxidative stress in OTA-induced cytotoxicity and apoptosis. In addition, our results are the first time to show that selenomethionine protects cells from OTA-induced cytotoxicity and apoptosis by improving selenoenzyme expression and by promoting antioxidant capacity. Therefore, selenomethionine supplementation may be an attractive strategy for protecting humans and animals from the risk of kidney damage caused by OTA.

## Supporting Information

S1 Table Primers Used for Real-Time Quantitative PCR. (DOC)
