# Supplementary material for: Selenium Alleviates Porcine Nephrotoxicity of Ochratoxin A by Improving Selenoenzyme Expression In Vitro
Source: PLoS One. 2015 Mar 24;10(3):e0119808. doi: 10.1371/journal.pone.0119808 (PMC4372527; doi:10.1371/journal.pone.0119808)
Supplement: S1 Table — (DOC) [file pone.0119808.s001.doc]

**Supporting Information**

**S1 Table. Primers Used for Real-Time Quantitative PCR**

| Gene | Accession number | Primer sequence(5’–3’) | Product  (bp) |
| --- | --- | --- | --- |
| Actb | DQ845171.1 | Forward：CTGCGGCATCCACGAAACT | 147 |
|  |  | Reverse：AGGGCCGTGATCTCCTTCTG |  |
| GPx1 | NM_214201 | Forward：TGGGGAGATCCTGAATTG | 172 |
|  |  | Reverse：GATAAACTTGGGGTCGGT |  |
| GPx4 | NM_214407.1 | Forward: GATTCTGGCCTTCCCTTGC | 183 |
|  |  | Reverse: TCCCCTTGGGCTGGACTTT |  |
| TR1 | NM_214154 | Forward: CCCTGGTGACAAAGAGTA | 184 |
|  |  | Reverse: GTCCTGGTCAAATCCTCT |  |
